# Electroconvulsive Therapy and Time to Psychiatric Readmission in Schizophrenia

**DOI:** 10.1111/acps.70115

**Published:** 2026-06-05

**Authors:** Linnea Stenmark, Mussie Msghina, Axel Nordenskjöld

**Affiliations:** ^1^ Faculty of Medicine and Health Örebro University Örebro Sweden; ^2^ Department of Clinical Neuroscience Karolinska Institutet Stockholm Sweden

**Keywords:** electroconvulsive therapy, patient readmission, schizophrenia

## Abstract

**Introduction:**

There is evidence that electroconvulsive therapy (ECT) can augment the effects of antipsychotics in schizophrenia. However, there is limited evidence from Western populations on whether ECT reduces the risk of relapse compared to non‐ECT treatment in schizophrenia. This study aimed to compare time to psychiatric readmission or suicide among inpatients with schizophrenia treated with or without ECT, as well as those receiving index series with or without continuation ECT (C‐ECT).

**Methods:**

Register‐based study utilizing data from Swedish nationwide registers. Patients with schizophrenia who received both inpatient ECT and non‐ECT treatment between 2011 and 2020 were included. Each patient served as their own control, contributing one admission with and without ECT. Admissions with or without ECT, as well as index series with or without C‐ECT, were compared in univariate Cox regression analyses regarding time to psychiatric readmission or suicide within 30 days, 90 days, 180 days, and 1 year from discharge.

**Results:**

A total of 351 patients were included. Of these patients, 64.1% were male and the mean (SD) age was 48.2 (14.4) years. ECT was associated with longer time to readmission or suicide compared to non‐ECT treatment within 30 days (HR: 0.66; 95% CI: 0.50–0.87; *p* = 0.003), 90 days (HR: 0.70; 95% CI: 0.56–0.87; *p* = 0.001), 180 days (HR: 0.68; 95% CI: 0.56–0.82; *p* = < 0.001), and 1 year from discharge (HR: 0.77; 95% CI: 0.65–0.91; *p* = 0.003). Furthermore, 15 patients received C‐ECT, which was associated with longer time to readmission or suicide compared to index series without C‐ECT within 90 days (HR: 0.13; 95% CI: 0.02–0.91; *p* = 0.040) and 180 days from discharge (HR: 0.26; 95% CI: 0.08–0.81; *p* = 0.021).

**Conclusion:**

This study suggests that ECT can be associated with a longer time to relapse compared to non‐ECT treatment in schizophrenia.

## Summary

- Significant outcomes
  ○ ECT was associated with longer time to relapse compared to non‐ECT treatment within 30 days, 90 days, 180 days, and 1 year from discharge in schizophrenia.
  ○ It may be particularly beneficial to try ECT in those with an insufficient response to three or more antipsychotics or clozapine.
  ○ A more favorable outcome was observed in the small number of patients treated with C‐ECT compared to index series without C‐ECT.
- Limitations
  ○ Some factors associated with relapse in schizophrenia were not accessible in register data. The study participants' non‐ECT admissions served as the control group to improve comparability between admissions with or without ECT.
  ○ Differences in length of stay and admission order may have influenced the results.
  ○ Few patients who received C‐ECT were included in the study.

## 1 Introduction

The lifetime risk of schizophrenia is estimated at 0.7% [1]. Most patients relapse within 5 years after having responded to treatment for first‐episode schizophrenia or schizoaffective disorder. Thereafter, the rate of relapse continues to be high [2, 3, 4]. Electroconvulsive therapy (ECT) could be considered for patients with an insufficient response to clozapine, as well as for patients with acute severe psychotic symptoms that are difficult to manage, catatonia, or a significant suicide risk according to the American Psychiatric Association. Those who respond to ECT could be considered for continuation ECT (C‐ECT) [5]. There is evidence that ECT can augment the effects of antipsychotics in schizophrenia. A meta‐analysis of 11 randomized controlled trials (RCT) found that ECT, combined with any non‐clozapine antipsychotic drug, was more effective than non‐clozapine antipsychotics alone in patients with an insufficient response to antipsychotics [6]. A meta‐analysis of 18 RCTs found that ECT, combined with clozapine, was more effective than clozapine alone in patients with an insufficient response to clozapine [7].

There is limited evidence whether ECT reduces the risk of readmission compared to non‐ECT treatment in schizophrenia. Lin et al. [8] matched 2.074 patients with schizophrenia who received inpatient ECT to the same number of inpatients who received non‐ECT treatment. The rate of readmission within 1 year after discharge was significantly lower compared to the year before admission for those treated with ECT. The difference was not statistically significant for patients who received non‐ECT treatment. Ying et al. [9] used propensity score matching to compare 570 patients with schizophrenia and related disorders who received inpatient ECT to 369 patients who received inpatient non‐ECT treatment regarding rates of readmission within 3 and 6 months from discharge. The study found that ECT was associated with significantly lower rates of readmission compared to non‐ECT treatment. Han et al. [10] studied 7.724 inpatients with mixed psychiatric disorders, of whom 2.065 patients (26.7%) received ECT and 3.448 patients (44.6%) were diagnosed with schizophrenia and related disorders. ECT was found to be significantly associated with reduced risk of 30‐day readmission compared to non‐ECT treatment. By contrast, Ma et al. [11] included 13.831 inpatients with mixed psychiatric disorders, of whom 2.460 patients (17.8%) received ECT and 4.801 patients (34.7%) were diagnosed with schizophrenia spectrum disorders. The study found no significant relationship between ECT and readmission rates within 6 months of discharge.

Previous studies have mainly included Asian inpatients [8, 9, 10, 11]. However, ECT utilization [12], as well as access to and prescription patterns of antipsychotics, vary significantly worldwide [13]. Schizophrenia constitutes the most common indication for ECT in Asia, whereas it is a less frequent indication in Western countries [12, 14]. Furthermore, ECT is more commonly administered to Asian inpatients compared to their Western counterparts [12]. Thus, there is a need for further studies investigating whether ECT reduces the risk of relapse compared to non‐ECT treatment in schizophrenia within a Western context.

### 1.1 Aims

To determine whether ECT is associated with longer or shorter time to psychiatric readmission or suicide compared to non‐ECT treatment in schizophrenia and whether C‐ECT is associated with longer or shorter time to psychiatric readmission or suicide compared to index series without C‐ECT. The secondary aim was to explore differential associations of ECT compared to non‐ECT treatment across subgroups of the study population.

## 2 Materials and Methods

### 2.1 Study Design

This study was based on data from multiple Swedish nationwide registers. Adult patients with schizophrenia who received both inpatient ECT and inpatient non‐ECT treatment in Sweden between January 1, 2011, and December 31, 2020, were included. Therefore, patients had to have a minimum of two hospitalizations during this period. Each patient contributed to one admission with and without ECT. ECT admissions were compared to non‐ECT admissions. The outcome was time to psychiatric readmission or suicide within 30 days, 90 days, 180 days, and 1 year from discharge. The patients were followed until readmission, death, or the end of the study (December 31, 2021). Time to readmission or suicide was also compared between index series with or without C‐ECT. In the first sensitivity analysis, ECT admissions were matched to non‐ECT admissions by length of stay. In the second sensitivity analysis, admissions were stratified by whether the ECT admission occurred first or last for each patient. Subgroup analyses were conducted to identify differences in time to readmission or suicide between admissions with or without ECT in subgroups of the study population.

### 2.2 Data Sources

The National Patient Register includes information about admission and discharge dates, involuntary psychiatric care, and inpatient procedures such as ECT. The part of the register covering inpatient care has been highly complete since 1987 and is over 99% complete for psychiatric hospital discharges. Diagnoses are coded according to the Swedish version of the International Statistical Classification of Diseases and Related Health Problems, 10th Revision (ICD‐10‐SE) [15]. Inpatients with a main diagnosis of schizophrenia were identified through the code F20. Psychiatric comorbidity was also identified through codes (anxiety disorder: F40, F41, F43‐45, or F48; cannabis use disorder: F12; substance use disorder: F10‐F16, F18, or F19; major depressive disorder: F32, F33, or F34.1). Readmission during follow‐up with a main diagnosis of any psychiatric disorder (F01‐F99) was considered a psychiatric readmission.

The Swedish National Quality Register for ECT (Q‐ECT) provides information about symptom severity, ECT parameters, number of sessions, and response to treatment. All hospitals that provide ECT in Sweden report to the Q‐ECT [16]. Of all patients treated with ECT in 2020, 92.4% were included in the Q‐ECT [17].

The Longitudinal Integrated Database for Health Insurance and Labour Market Studies (LISA) contains information about demographics such as education level, employment status, and marital status. The LISA includes all Swedish citizens and residents aged 16 years and above since 1990, and those aged 15 years and above since 2010 [18].

The National Prescribed Drug Register provides information about all prescription drugs dispensed at Swedish pharmacies since 2005 [19].

The Multi‐Generation Register contains information about index persons, including all individuals born in Sweden after 1931 and those registered as Swedish residents since 1961. For those born in Sweden after 1949, the register is 100% complete for mothers and 98% complete for fathers [20]. The register was used to identify first‐degree relatives with psychotic disorders (ICD‐10‐SE: F20 or F22‐F29; ICD‐9‐SE: 295, 297, 298B, or 298E‐X; ICD‐8‐SE: 295, 297, 298.1, 298.3–298.9, or 299).

The National Cause of Death Register includes all deaths of Swedish residents since 1952. The underlying cause of death is recorded for 96% of individuals [21]. The register was used to identify deaths during follow‐up.

### 2.3 Electroconvulsive Therapy Setting

ECT is usually administered using bidirectional constant current brief pulse devices: MECTA Spectrum (MECTA Corp.) or Thymatron System IV (Somatics LLC) [22]. Study participants received a median of 7 sessions per treatment series (interquartile range (IQR): 5–10; range: 1–93). Electrode placement during the first session was unilateral in 177 patients (50.4%), bitemporal in 74 patients (21.1%), bifrontal in 9 patients (2.6%), and unknown in 91 patients (25.9%). Electrode placement during the last session was unilateral in 184 patients (52.4%), bitemporal in 65 patients (18.5%), bifrontal in 9 patients (2.6%), and unknown in 93 patients (26.5%). The mean ± standard deviation (SD) pulse width during the first session was 0.5 ± 0.1 ms, whereas the mean ± SD frequency was 62.0 ± 21.3 Hz, the mean ± SD duration was 6.7 ± 1.3 s, the mean ± SD current was 844.5 ± 50.7 mA, and the mean ± SD charge was 359.4 ± 163.1 mC.

### 2.4 Statistics

The characteristics of admissions with or without ECT were compared using the *χ* ^2^ test and the Mann‐Whitney *U*‐test. Data were analyzed in univariate Cox regression analyses. Admissions with or without ECT had to be in consecutive order, although the ECT admission could come either first or last. The first ECT admission between January 1, 2011 to December 31, 2020 with a non‐ECT admission directly before or after was selected. If the ECT admission had non‐ECT admissions both before and after, the non‐ECT admission was selected by generating random numbers. Index series with C‐ECT were defined as receiving ECT within 2 weeks from discharge. The risk of introducing immortal time in the C‐ECT group was considered. Therefore, readmission or suicide before the median time to start C‐ECT was censored for those receiving non‐ECT treatment or an index series without C‐ECT in some analyses. The univariate Cox regression model was applied after stratification of the study population in the second sensitivity analysis and subgroup analyses. Thereafter, interaction terms were added to the model to determine whether any differences were statistically significant. The significance level was set at 0.05. SAS software (SAS Institute Inc. Released 2013. Version 9.4. Cary, NC: SAS Institute Inc.) and SPSS (IBM Corp. Released 2017. IBM SPSS Statistics for Windows, Version 25.0. Armonk, NY: IBM Corp.) were used to perform data management and statistical analyses.

### 2.5 Ethical Considerations

This study has received approval from the Regional Ethical Review Board in Uppsala, Sweden (2014/174) and the Swedish Ethical Review Authority (2019‐06558). Pseudonymized data were collected from the registers. Study participants were not identifiable at any time. Thus, participants were not informed of the study nor asked to provide written consent.

## 3 Results

### 3.1 Participants

A total of 1.953 unique patients were admitted with a main diagnosis of schizophrenia during the study period. Of these, 98 patients had only one ECT admission and 14 patients had only multiple ECT admissions during the study period. A total of 631 patients had only one non‐ECT admission and 859 had only multiple non‐ECT admissions. These patients were excluded from the study due to the lack of a control group. Thus, a total of 351 unique patients with both ECT and non‐ECT admissions were included in this study. Study participants had a median of five hospitalizations during the study period (IQR: 3–10; range: 2–62).

The ECT admission came before the non‐ECT admission in 135 patients (38.5%). When the ECT admission occurred first, the median time to the non‐ECT admission was 141.0 days (IQR: 14.0–345.0), whereas when the non‐ECT admission occurred first, the median time to the ECT admission was 26.0 days (IQR: 3.0–173.3). The ECT admissions were more likely to be involuntary. Furthermore, the median length of stay was significantly longer for the ECT admissions (Table 1).

**TABLE 1:** Characteristics of admissions with and without ECT.

Characteristic	Admissions with ECT *n* = 351	Admissions without ECT *n* = 351	*p*
Demographics			
Male, *n* (%)	225 (64.1)	225 (64.1)	1.000
Age in years, mean (SD)	48.2 (14.5)	48.1 (14.3)	0.901
Married or widowed, *n* (%)	17 (4.8)	18 (5.1)	0.832
Cohabiting, *n* (%)	49 (14.0)	48 (13.7)	0.913
Low education level (less than high school), *n* (%)	109 (31.1)	107 (30.5)	0.993
Middle education level (high school), *n* (%)	186 (53.0)	187 (53.3)	
High education level (college), *n* (%)	49 (14.0)	49 (14.0)	
Employed, *n* (%)	28 (8.0)	25 (7.1)	0.668
Inpatient characteristics			
Involuntarily admitted, *n* (%)	185 (57.2)	128 (36.5)	< 0.001
Length of stay in days, median (IQR)	48.0 (26.0–86.0)	10.0 (3.0–32.0)	< 0.001
Prior psychiatric hospitalizations			
Number of prior psychiatric hospitalizations, median (IQR)	15.0 (6.0–29.0)	15.0 (7.0–29.0)	0.793
Years since first psychiatric admission, median (IQR)	22.0 (9.0–32.0)	22.0 (10.0–32.0)	0.992
Subtype			
Catatonic schizophrenia, *n* (%)	14 (4.0)	9 (2.6)	0.298
Paranoid schizophrenia, *n* (%)	163 (46.4)	166 (47.3)	0.821
Other subtypes, *n* (%)	174 (49.6)	176 (50.1)	0.880
Psychiatric comorbidity			
Anxiety disorder, *n* (%)	96 (27.4)	95 (27.1)	0.932
Cannabis use disorder, *n* (%)	24 (6.8)	24 (6.8)	1.000
Substance use disorder, *n* (%)	59 (16.8)	59 (16.8)	1.000
Major depressive disorder, *n* (%)	88 (25.1)	86 (24.5)	0.861
Antipsychotics			
Total number of antipsychotics ever collected, median (IQR)	3.0 (2.0–5.0)	3.0 (2.0–5.0)	1.000
Long‐acting injectable antipsychotics^a^, *n* (%)	118 (33.6)	110 (31.3)	0.519
Clozapine^a^, *n* (%)	116 (33.0)	111 (31.6)	0.687
Other pharmacological agents			
Antidepressants^a^, *n* (%)	148 (42.2)	148 (42.2)	1.000
Anxiolytics (benzodiazepines excluded)^a^, *n* (%)	53 (15.1)	46 (13.1)	0.448
Benzodiazepines^a^, *n* (%)	146 (41.6)	144 (41.0)	0.878
Biperiden^a^, *n* (%)	60 (17.1)	64 (18.2)	0.692
Mood stabilizers^a^, *n* (%)	56 (16.0)	53 (15.1)	0.755
Family history in first‐degree relatives			
Family history of psychotic disorders, *n* (%)	39 (11.1)	40 (11.4)	0.905

Abbreviations: ECT, electroconvulsive therapy; IQR, interquartile range; SD, standard deviation.

^a^ Antipsychotics in tablet form collected from a pharmacy within 100 days before admission or long‐acting injectable antipsychotics collected from a pharmacy within 180 days before admission.

Almost half of the study participants had prior experience of treatment with clozapine, 47.3% of patients treated with ECT and 46.4% of patients treated without ECT. There was no significant difference in the proportion of study participants who initiated clozapine or long‐acting injectable (LAI) antipsychotics following the ECT admissions compared to the non‐ECT admissions. Out of 235 patients not treated with clozapine within 100 days before admission, 71 patients (30.2%) had collected clozapine within 100 days following their ECT admission. The corresponding proportion was 77 out of 240 patients (32.1%) following their non‐ECT admission (*p* = 0.670). Out of 233 patients not treated with LAI antipsychotics within 180 days before admission, 94 patients (40.3%) had collected a LAI antipsychotic within 180 days following their ECT admission, whereas 99 out of 241 patients (41.3%) had collected a LAI antipsychotic following their non‐ECT admission (*p* = 0.870).

A total of four study participants died during follow‐up. Of these, three deaths were due to suicide. The ECT admissions had one subsequent suicide and the non‐ECT admissions were followed by two suicides. There were no statistically significant differences in ratings on the Clinical Global Impression—Severity (CGI‐S) scale before starting ECT and the Clinical Global Impression—Improvement (CGI‐I) scale within 1 week after ending ECT between index series with or without C‐ECT (Table 2). The median time to start C‐ECT was 6 days from discharge (IQR: 4–10).

**TABLE 2:** Clinical global impression—severity ratings before starting ECT and clinical global impression—improvement ratings within 1 week after ending ECT.

Rating scale	Index series without C‐ECT *n* = 336	Index series with C‐ECT *n* = 15	*p*
CGI‐S before starting ECT			
Mildly ill, *n* (%)	2 (0.6)	0 (0.0)	0.870
Moderately ill, *n* (%)	16 (4.8)	0 (0.0)	
Markedly ill, *n* (%)	75 (22.3)	5 (33.3)	
Severely ill, *n* (%)	106 (31.5)	5 (33.3)	
Among the most extremely ill patients, *n* (%)	35 (10.4)	1 (6.7)	
Not assessed, *n* (%)	102 (30.4)	4 (26.7)	
CGI‐I within 1 week after ending ECT			
Very much improved, *n* (%)	42 (12.5)	3 (20.0)	0.667
Much improved, *n* (%)	109 (32.4)	7 (46.7)	
Minimally improved, *n* (%)	49 (14.6)	1 (6.7)	
No change, *n* (%)	14 (4.2)	0 (0.0)	
Minimally worse, *n* (%)	0 (0.0)	0 (0.0)	
Much worse, *n* (%)	3 (0.9)	0 (0.0)	
Very much worse, *n* (%)	0 (0.0)	0 (0.0)	
Not assessed, *n* (%)	119 (35.4)	4 (26.7)	

Abbreviations: CGI‐I, Clinical Global Impression—Improvement; CGI‐S, Clinical Global Impression—Severity; C‐ECT, continuation electroconvulsive therapy; ECT, electroconvulsive therapy.

### 3.2 Readmission Rates

Readmission rates of the ECT admissions were 23.9%, 40.7%, 52.7%, and 70.1% within 30 days, 90 days, 180 days, and 1 year from discharge. Readmission rates of the non‐ECT admissions were 34.5%, 52.4%, 67.0%, and 76.4% within 30 days, 90 days, 180 days, and 1 year from discharge. Readmission rates of the 15 study participants who received C‐ECT were 6.7%, 6.7%, 20.0%, and 73.3% within 30 days, 90 days, 180 days, and 1 year from discharge.

### 3.3 Time to Readmission or Suicide

Treatment with ECT was associated with longer time to readmission or suicide compared to non‐ECT treatment within 30 days (hazard ratio (HR): 0.66; 95% confidence interval (CI): 0.50–0.87; *p* = 0.003), 90 days (HR: 0.70; 95% CI: 0.56–0.87; *p* = 0.001), 180 days (HR: 0.68; 95% CI: 0.56–0.82; *p* = < 0.001), and 1 year from discharge (HR: 0.77; 95% CI: 0.65–0.91; *p* = 0.003) (Figure S1).

Treatment with C‐ECT was associated with longer time to readmission or suicide compared to index series without C‐ECT within 90 days (HR: 0.13; 95% CI: 0.02–0.91; *p* = 0.040) and 180 days from discharge (HR: 0.26; 95% CI: 0.08–0.81; *p* = 0.021). The difference was not statistically significant within 30 days (HR: 0.27; 95% CI: 0.04–1.93; *p* = 0.192) or 1 year from discharge (HR: 0.76; 95% CI: 0.42–1.38; *p* = 0.360). Readmission or suicide before the median time to start C‐ECT was censored in this analysis.

**FIGURE 1:**
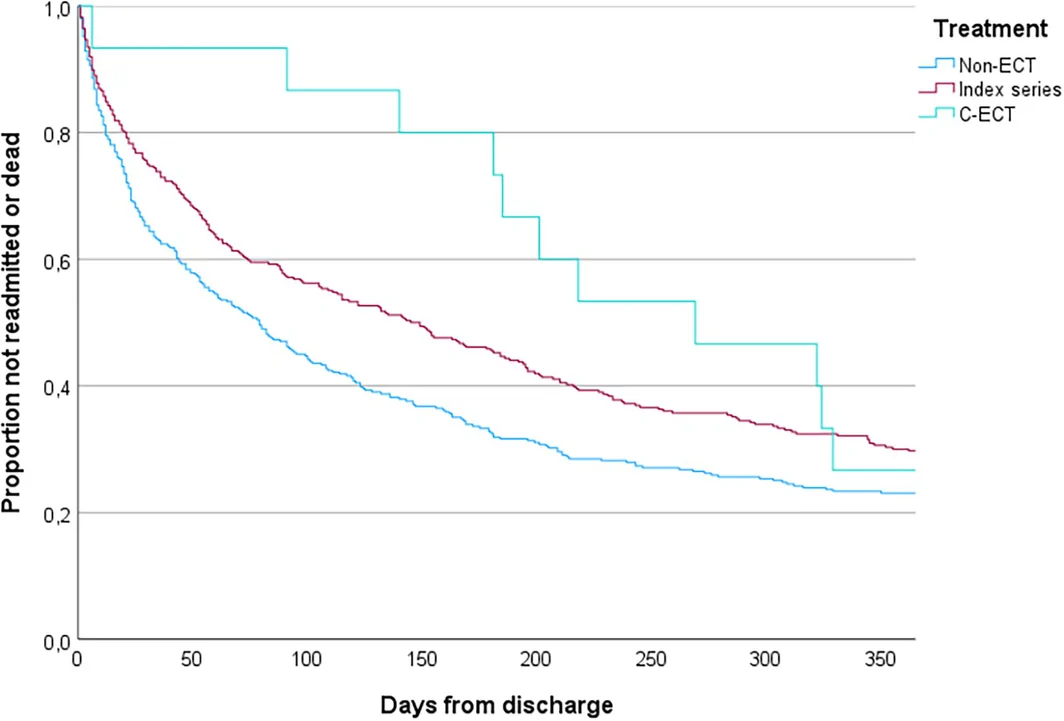
Kaplan–Meier curve of time to readmission or suicide after discharge in patients who received non‐ECT treatment or an index series with or without C‐ECT. C‐ECT, continuation electroconvulsive therapy; ECT, electroconvulsive therapy.

Univariate Cox regression analyses showed that these associations were still statistically significant when including non‐ECT treatment and index series with and without C‐ECT in the analysis (Figure 1 and Table 3).

**TABLE 3:** Univariate Cox regression analysis of the association between treatment and time to readmission or suicide.

Time from discharge	HR (95% CI)	*p*
Within 30 days		
Non‐ECT treatment^a^	Reference	
Index series without C‐ECT^a^	0.63 (0.45–0.87)	**0.006**
Index series with C‐ECT	0.22 (0.03–1.57)	0.131
Within 90 days		
Non‐ECT treatment^a^	Reference	
Index series without C‐ECT^a^	0.71 (0.56–0.90)	**0.005**
Index series with C‐ECT	0.11 (0.02–0.78)	**0.027**
Within 180 days		
Non‐ECT treatment^a^	Reference	
Index series without C‐ECT^a^	0.69 (0.56–0.85)	**< 0.001**
Index series with C‐ECT	0.22 (0.07–0.68)	**0.009**
Within 1 year		
Non‐ECT treatment^a^	Reference	
Index series without C‐ECT^a^	0.77 (0.64–0.92)	**0.005**
Index series with C‐ECT	0.66 (0.36–1.21)	0.180

*Note:* The threshold for statistical significance was set at 0.05.

Abbreviations: CI, confidence interval; C‐ECT, continuation electroconvulsive therapy; ECT, electroconvulsive therapy; HR, hazard ratio.

^a^ Readmission or suicide before the median time to start C‐ECT was censored for those receiving non‐ECT treatment or an index series without C‐ECT.

### 3.4 First Sensitivity Analysis

A total of 110 ECT admissions were matched to 110 non‐ECT admissions by length of stay. For both the ECT and non‐ECT admissions, 24 admissions (21.8%) were ≤ 14 days, 11 admissions (10.0%) were between 15 and 30 days, and 75 admissions (68.2%) were ≥ 31 days. There were only four study participants who received C‐ECT in this analysis. The median length of stay for the ECT and non‐ECT admissions were 44.5 days (IQR: 20.3–83.8) and 41.0 days (IQR:16.8–75.3), respectively (*p* = 0.395). In univariate Cox regression analyses, differences in time to readmission or suicide between ECT admissions and non‐ECT admissions were not statistically significant within 30 days (HR: 0.72; 95% CI: 0.43–1.20; *p* = 0.205), 90 days (HR: 0.73; 95% CI: 0.50–1.08; *p* = 0.119), 180 days (HR: 0.78; 95% CI: 0.55–1.10; *p* = 0.152), or 1 year from discharge (HR: 0.88; 95% CI: 0.64–1.20; *p* = 0.408) (Figure S2).

### 3.5 Second Sensitivity Analysis

The ECT admission came before the non‐ECT admission in 155 patients (38.5%), whereas the non‐ECT admission came before the ECT admission in 216 patients (61.5%). There was a significant interaction between admission order and inpatient treatment. ECT admissions were associated with longer time to readmission or suicide compared to non‐ECT admissions when the non‐ECT admission occurred first, but not when the ECT admission occurred first (Table S1).

### 3.6 Subgroup Analyses

Study participants who had previously tried three to five antipsychotics had markedly superior outcomes within 30 days and 90 days from discharge when treated with ECT compared to non‐ECT treatment, but there was no statistically significant difference in time to readmission or suicide in those who had previously tried zero to two antipsychotics. Similarly, patients treated with clozapine within 100 days before admission had superior outcomes within 90 days from discharge when treated with ECT compared to non‐ECT treatment, but those treated without clozapine before admission did not have significantly better outcomes when treated with ECT compared to non‐ECT treatment (Tables S2 and S3).

## 4 Discussion

The main analysis of this study suggests that ECT is associated with longer time to relapse compared to non‐ECT treatment in schizophrenia. The difference in time to readmission or suicide was statistically significant within 30 days, 90 days, 180 days, and 1 year from discharge. Nevertheless, suicide comprised a very small proportion of the outcome. There were only three study participants who died by suicide during follow‐up. Therefore, the outcome should almost exclusively be interpreted as readmission.

The few studies that have investigated whether ECT reduces the risk of readmission in schizophrenia have reported differing results and have mainly included Asian inpatients [8, 9, 10, 11]. Furthermore, some of these studies have included patients with mixed psychiatric disorders without specifically reporting the outcome for patients with schizophrenia nor the ECT setting [10, 11], thereby limiting comparability. The methodology of Lin et al. [8] exhibits similarities to the present study as the 2.074 inpatients treated with ECT also served as the control group as readmission rates within 1 year before and after an ECT admission were compared. Ying et al. [9] used propensity score matching to improve comparability between ECT and non‐ECT admissions. Both studies reported results that are in congruence with those of the present study.

One study reported that patients treated with ECT tend to be more severely ill compared to inpatients receiving non‐ECT treatment [23]. In the present study, the characteristics of admissions with or without ECT were similar. The only statistically significant differences were that the ECT admissions were more likely to be involuntary and had a longer length of stay. These differences could indicate that study participants were treated with ECT when they had more severe symptoms, which has been associated with an increased risk of relapse in schizophrenia [24, 25], as well as not being in remission at discharge [24]. Moreover, studies have found involuntary admissions to be associated with increased readmission risk compared to voluntary admissions [26, 27, 28]. If the ECT admissions had an inherently higher readmission risk compared to the non‐ECT admissions, the true effects of ECT could have been underestimated in the present study.

Previous studies have found a shorter length of stay to be associated with increased readmission risk in schizophrenia. A likely explanation for this could be the presence of more residual symptoms at discharge [29]. In the first sensitivity analysis, the ECT and non‐ECT admissions were matched by length of stay. There was no statistically significant difference between the groups after matching. Although the hazard ratio point estimates were similar to those in the main analysis, especially within 30 days, 90 days, and 180 days from discharge, statistical significance was not reached. This could possibly be explained by insufficient power as there were only 110 patients in this analysis compared to 351 patients in the main analysis. Nevertheless, the longer length of stay observed in the ECT admissions relative to the non‐ECT admissions may have influenced the results of the main analysis, as extended hospitalization may promote clinical stabilization by allowing optimization of pharmacological treatment, nursing care, and discharge planning.

In the second sensitivity analysis, a significant interaction was observed between admission order and inpatient treatment. One possible explanation is that study participants received ECT during exacerbations of symptoms despite optimized pharmacological treatment. For some patients, ECT may have been effective, resulting in a prolonged time to readmission. In such cases, ECT would likely be administered again during subsequent admissions, therefore no subsequent non‐ECT admission would be available for inclusion in this study. Conversely, when an ECT admission was followed by a non‐ECT admission, this clinical decision may have reflected a poor response to ECT. This non‐ECT admission would be available for inclusion in this study. Thus, one interpretation of the findings is that ECT may be effective following pharmacological failure; however, if ECT does not result in significant improvement, it is unlikely to be superior to pharmacological treatment.

Few interaction terms were statistically significant in the subgroup analyses. This could be explained by the fact that most subgroups had favorable outcomes when treated with ECT, and therefore, differences were too small to be statistically significant. However, in several of these analyses, subgroups with more favorable outcomes comprised patients who had previously been treated with three to five antipsychotics or clozapine within 100 days prior to admission, suggesting a particularly poor response to antipsychotic treatment. These results indicate that ECT could be a particularly suitable choice for most patients with schizophrenia suffering from acute severe symptoms that are difficult to manage or are insufficiently responsive to antipsychotics.

Rates of readmission were high following both ECT and non‐ECT admissions. For the ECT admissions, 23.9%, 40.7%, 52.7%, and 70.1% were readmitted within 30 days, 90 days, 180 days, and 1 year from discharge. A recent meta‐analysis of 29 studies found relapse or readmission rates after ECT to be 24%, 40%, 41%, and 55% within 3 months, 6 months, 12 months, and 24 months from discharge. The studies included in the meta‐analysis, ranging from RCTs to small observational studies, differed considerably in their reported relapse or readmission rates. Relapse or readmission was most common within 3 months from discharge [30], which is in accordance with the results of the present study.

There is some evidence that C‐ECT can help maintain response or remission and prevent relapse in schizophrenia [31, 32]. There were only 15 study participants who received C‐ECT in this study, resulting in low statistical power with wide confidence intervals regarding the estimated effects of treatment. These patients were most likely responsive to the initial index series. C‐ECT was associated with longer time to readmission or suicide compared to index series without C‐ECT within 90 days and 180 days from discharge in this study, but statistical significance was not reached within 30 days or 1 year from discharge, most likely because of insufficient power.

This study is, to the best of our knowledge, the largest of its kind conducted within a Western context. It included patients treated in clinical practice at all hospitals in Sweden and utilized data from multiple Swedish nationwide registers with high degrees of completeness and reliability [15, 16, 17, 18, 19, 20, 21]. Nevertheless, this study has various limitations. Patients treated with ECT tend to have more severe symptoms compared to inpatients receiving non‐ECT treatment [23]. The study participants' non‐ECT admissions served as the control group to improve comparability between admissions with or without ECT. However, there were still some differences between ECT admissions and non‐ECT admissions, which indicate that study participants had more severe symptoms when treated with ECT. Differences in length of stay and admission order may have influenced the results of the main analysis. Some factors associated with relapse or readmission in schizophrenia, such as attitude toward treatment, medication side effects [25], or medication adherence [24], were not accessible in register data. Nonetheless, it could be argued that differences in such factors are likely smaller within an individual over time than between different individuals. Participants were considered to be treated with antipsychotics or other pharmacological agents if the patient had collected these drugs at a pharmacy. The register data did not contain information about symptom severity as assessed by rating scales for the non‐ECT admissions. This study included few patients who received C‐ECT, and although these patients had overall favorable outcomes compared to non‐ECT treatment or index series without C‐ECT, our results have some statistical uncertainty and should be interpreted cautiously.

## 5 Conclusions

This study suggests that ECT can be associated with longer time to relapse compared to non‐ECT treatment in schizophrenia. It may be particularly beneficial to try ECT in those with an insufficient response to three or more antipsychotics or clozapine. Further studies of the effects of ECT on rates of relapse, readmission, or suicide in schizophrenia are needed.

## Author Contributions

**Linnea Stenmark**, **Mussie Msghina**, **Axel Nordenskjöld:** conceptualization. **Linnea Stenmark**, **Mussie Msghina**, **Axel Nordenskjöld:** methodology. **Linnea Stenmark**, **Axel Nordenskjöld:** software. **Linnea Stenmark**, **Axel Nordenskjöld:** validation. **Linnea Stenmark**, **Axel Nordenskjöld:** formal analysis. **Linnea Stenmark**, **Axel Nordenskjöld:** investigation. **Axel Nordenskjöld:** resources. **Linnea Stenmark**, **Axel Nordenskjöld:** data curation. **Linnea Stenmark**, **Mussie Msghina**, **Axel Nordenskjöld:** writing – original draft. **Linnea Stenmark**, **Mussie Msghina**, **Axel Nordenskjöld:** writing – review and editing. **Linnea Stenmark**, **Axel Nordenskjöld:** visualization. **Axel Nordenskjöld:** supervision. **Linnea Stenmark**, **Axel Nordenskjöld:** project administration. **Axel Nordenskjöld:** funding acquisition.

## Funding

This work was supported by Region Örebro and Nyckelfonden at Örebro University Hospital (grant number 961230).

## Ethics Statement

This study has received approval from the Regional Ethical Review Board in Uppsala, Sweden (2014/174) and the Swedish Ethical Review Authority (2019‐06558). Study participants were not identifiable at any time as pseudonymized data were collected from the registers.

## Consent

Thus, participants were not informed of the study nor asked to provide written consent.

## Conflicts of Interest

The authors declare no conflicts of interest.

## Supporting information


**Figure S1:** Kaplan–Meier curve of time to readmission or suicide after discharge in patients treated with or without ECT.
**Figure S2:** Kaplan–Meier curve of time to readmission or suicide after discharge in patients treated with or without ECT in a sample matched by length of stay.
**Table S1:** Univariate Cox regression analysis of the association between whether the ECT admission came first or last and time to readmission or suicide.
**Table S2:** Univariate Cox regression analyses of the association between ECT and time to readmission or suicide within 30 days and 90 days from discharge, stratified by different subgroups of the study population with interaction term *p*‐values.
**Table S3:** Univariate Cox regression analyses of the association between ECT and time to readmission or suicide within 180 days and 1 year from discharge, stratified by different subgroups of the study population with interaction term *p*‐values.

## Data Availability

The data that support the findings of this study are available on request from the corresponding author. The data are not publicly available due to privacy or ethical restrictions.
