# Supplementary material for: Electroconvulsive Therapy and Time to Psychiatric Readmission in Schizophrenia
Source: Acta Psychiatr Scand. 2026 Jun 5;154(4):277–85. doi: 10.1111/acps.70115 (PMC13532970; doi:10.1111/acps.70115)
Supplement: Supplementary file 1 [file ACPS-154-277-s001.docx]

# Supplementary Material

# No. at risk

Non-ECT 351	203  155  129  109  95	89	81	81

ECT	351	243  202  178  151  131  121  106 103

**Supplementary Figure 1**. Kaplan-Meier curve of time to readmission or suicide after discharge in patients treated with or without ECT.

Abbreviations: ECT, electroconvulsive therapy.

# No. at risk

Non-ECT 110	66  47  42  37  34	33	33	33

ECT	110	77	63  51  45  41	37  35  34

**Supplementary Figure 2**. Kaplan-Meier curve of time to readmission or suicide after discharge in patients treated with or without ECT in a sample matched by length of stay.

Abbreviations: ECT, electroconvulsive therapy.

**Supplementary Table 1.** Univariate Cox regression analysis of the association between whether the ECT admission came first or last and time to readmission or suicide.

| **Time from discharge** | **HR (95% CI)** | **P-value** |
| --- | --- | --- |
| **Within 30 days** | | |
| ECT admission first | 1.20 (0.76-1.90) | 0.425 |
| Non-ECT admission first | 0.46 (0.32-0.66) | **<0.001** |
| **Within 90 days** | | |
| ECT admission first | 1.05 (0.73-1.50) | 0.800 |
| Non-ECT admission first | 0.55 (0.42-0.72) | **<0.001** |
| **Within 180 days** | | |
| ECT admission first | 1.03 (0.75-1.41) | 0.856 |
| Non-ECT admission first | 0.53 (0.41-0.67) | **<0.001** |
| **Within one year** | | |
| ECT admission first | 1.26 (0.96-1.67) | 0.100 |
| Non-ECT admission first | 0.56 (0.45-0.70) | **<0.001** |

Abbreviations: CI, confidence interval; ECT, electroconvulsive therapy; HR, hazard ratio.

Readmission rates following a first ECT admission were 28.9%, 43.7%, 57.8%, and 80.0% within 30 days, 90 days, 180 days, and one year from discharge. Readmission rates following a first non-ECT admission were 40.3%, 58.3%, 72.7%, and 82.4% within 30 days, 90 days, 180 days, and one year from discharge.

**Supplementary Table 2.** Univariate Cox regression analyses of the association between ECT and time to readmission or suicide within 30 days and 90 days from discharge, stratified by different subgroups of the study population with interaction term P-values.

| **Subgroup** | **30 days**  **HR (95% CI)** | **P-value** | **Interaction P-value** | **90 days**  **HR (95% CI)** | **P-value** | **Interaction P-value** |
| --- | --- | --- | --- | --- | --- | --- |
| **Sex** | | | | | | |
| Male | 0.65 (0.46-0.92) | **0.015** | 0.855 | 0.65 (0.49-0.86) | **0.002** | 0.350 |
| Female | 0.68 (0.43-1.08) | 0.100 | Reference | 0.80 (0.56-1.13) | 0.198 | Reference |
| **Age** | | | | | | |
| 18-30 years | 0.80 (0.38-1.69) | 0.552 | Reference | 0.83 (0.48-1.42) | 0.493 | Reference |
| 31-40 years | 0.54 (0.25-1.17) | 0.119 | 0.491 | 0.54 (0.30-0.96) | **0.034** | 0.296 |
| 41-50 years | 0.72 (0.42-1.23) | 0.230 | 0.842 | 0.71 (0.45-1.11) | 0.135 | 0.627 |
| 51-60 years | 0.56 (0.33-0.94) | **0.028** | 0.461 | 0.62 (0.41-0.94) | **0.024** | 0.377 |
| >60 years | 0.75 (0.39-1.46) | 0.400 | 0.947 | 0.88 (0.53-1.45) | 0.609 | 0.892 |
| **Inpatient characteristics** | | | | | | |
| Voluntarily admitted | 0.59 (0.40-0.85) | **0.005** | Reference | 0.68 (0.50-0.91) | **0.009** | Reference |
| Involuntarily admitted | 0.85 (0.55-1.33) | 0.481 | 0.208 | 0.76 (0.55-1.07) | 0.112 | 0.571 |
| Length of stay  ≤ 14 days | 1.07 (0.60-1.88) | 0.824 | Reference | 1.10 (0.69-1.76) | 0.680 | Reference |
| Length of stay  15-30 days | 0.66 (0.33-1.31) | 0.233 | 0.301 | 0.67 (0.39-1.17) | 0.158 | 0.177 |
| Length of stay  >30 days | 0.77 (0.49-1.22) | 0.270 | 0.385 | 0.74 (0.53-1.05) | 0.092 | 0.188 |
| **Subtype** | | | | | | |
| No paranoid schizophrenia | 0.64 (0.44-0.92) | **0.017** | Reference | 0.64 (0.48-0.87) | **0.004** | Reference |
| Paranoid schizophrenia | 0.69 (0.46-1.05) | 0.084 | 0.750 | 0.77 (0.56-1.06) | 0.114 | 0.384 |
| **Psychiatric comorbidity** | | | | | | |
| No anxiety disorder | 0.61 (0.45-0.85) | **0.003** | Reference | 0.69 (0.53-0.89) | **0.004** | Reference |
| Anxiety disorder | 0.82 (0.47-1.41) | 0.463 | 0.377 | 0.74 (0.49-1.11) | 0.147 | 0.755 |
| No cannabis use disorder | 0.67 (0.50-0.89) | **0.005** | Reference | 0.72 (0.57-0.90) | **0.004** | Reference |
| Cannabis use disorder | 0.50 (0.12-1.99) | 0.322 | 0.680 | 0.45 (0.18-1.14) | 0.091 | 0.390 |
| No substance use disorder | 0.61 (0.45-0.83) | **0.001** | Reference | 0.67 (0.53-0.85) | **0.001** | Reference |
| Substance use disorder | 1.03 (0.50-2.10) | 0.946 | 0.193 | 0.85 (0.51-1.43) | 0.538 | 0.392 |
| No major depressive disorder | 0.68 (0.50-0.93) | **0.015** | Reference | 0.65 (0.51-0.84) | **<0.001** | Reference |
| Major depressive disorder | 0.60 (0.33-1.10) | 0.100 | 0.693 | 0.88 (0.57-1.37) | 0.574 | 0.234 |
| **Prior psychiatric hospitalizations** | | | | | | |
| 0-5 prior hospitalizations | 0.63 (0.30-1.33) | 0.223 | 0.529 | 0.69 (0.40-1.18) | 0.172 | 0.501 |
| 6-15 prior hospitalizations | 0.62 (0.38-1.00) | 0.050 | 0.374 | 0.61 (0.42-0.89) | **0.010** | 0.179 |
| 16-25 prior hospitalizations | 0.49 (0.25-0.94) | **0.033** | 0.188 | 0.60 (0.35-1.02) | 0.058 | 0.227 |
| >25 prior hospitalizations | 0.83 (0.52-1.34) | 0.448 | Reference | 0.87 (0.60-1.26) | 0.454 | Reference |
| **Years since first psychiatric admission** | | | | | | |
| ≤10 years | 0.66 (0.50-0.87) | **0.003** | Reference | 0.70 (0.56-0.87) | **0.001** | Reference |
| 11-25 years | 0.72 (0.44-1.19) | 0.202 | 0.852 | 0.69 (0.47-1.01) | 0.056 | 0.585 |
| >25 years | 0.62 (0.41-0.92) | **0.018** | 0.837 | 0.66 (0.47-0.92) | **0.013** | 0.462 |
| **Total number antipsychotics ever collected** | | | | | | |
| 0-2 antipsychotics | 0.95 (0.61-1.47) | 0.806 | Reference | 0.93 (0.65-1.32) | 0.679 | Reference |
| 3-5 antipsychotics | 0.47 (0.30-0.72) | **<0.001** | **0.026** | 0.55 (0.39-0.77) | **<0.001** | **0.035** |
| >5 antipsychotics | 0.70 (0.37-1.34) | 0.285 | 0.439 | 0.71 (0.44-1.16) | 0.173 | 0.386 |
| **Antipsychotics collected within 100-180 days before admission^a^** | | | | | | |
| No long-acting injectable antipsychotics | 0.73 (0.53-1.01) | 0.060 | Reference | 0.74 (0.57-0.96) | **0.024** | Reference |
| Long-acting injectable antipsychotics | 0.52 (0.30-0.89) | **0.017** | 0.262 | 0.63 (0.42-0.92) | **0.019** | 0.500 |
| No clozapine | 0.81 (0.57-1.15) | 0.240 | Reference | 0.85 (0.65-1.11) | 0.240 | Reference |
| Clozapine | 0.45 (0.29-0.72) | **<0.001** | 0.056 | 0.47 (0.33-0.68) | **<0.001** | **0.012** |
| **Other pharmacological agents collected within 100 days before admission** | | | | | | |
| No antidepressants before | 0.82 (0.57-1.18) | 0.288 | Reference | 0.74 (0.56-0.98) | **0.036** | Reference |
| Antidepressants before | 0.49 (0.32-0.76) | **0.001** | 0.084 | 0.65 (0.46-0.91) | **0.012** | 0.565 |
| No anxiolytics before | 0.68 (0.51-0.92) | **0.013** | Reference | 0.72 (0.57-0.91) | **0.006** | Reference |
| Anxiolytics before | 0.54 (0.26-1.13) | 0.099 | 0.579 | 0.58 (0.34-1.00) | **0.049** | 0.553 |
| No benzodiazepines before | 0.68 (0.46-1.00) | 0.050 | Reference | 0.72 (0.54-0.97) | **0.028** | Reference |
| Benzodiazepines before | 0.64 (0.43-0.95) | **0.027** | 0.847 | 0.67 (0.49-0.93) | **0.017** | 0.744 |
| No mood stabilizers before | 0.71 (0.53-0.96) | **0.028** | Reference | 0.72 (0.57-0.92) | **0.008** | Reference |
| Mood stabilizers before | 0.42 (0.20-0.88) | **0.020** | 0.222 | 0.57 (0.33-1.00) | **0.049** | 0.492 |
| **Family history in first-degree relatives** | | | | | | |
| No family history of psychotic disorders | 0.67 (0.50-0.91) | **0.010** | Reference | 0.70 (0.56-0.89) | **0.003** | Reference |
| Family history of psychotic disorders | 0.59 (0.29-1.19) | 0.138 | 0.735 | 0.68 (0.37-1.25) | 0.216 | 0.875 |

Abbreviations: CI, confidence interval; ECT, electroconvulsive therapy; HR, hazard ratio.

^a^Antipsychotics in tablet form collected within 100 days before admission or long-acting injectable antipsychotics collected within 180 days before admission.

**Supplementary Table 3.** Univariate Cox regression analyses of the association between ECT and time to readmission or suicide within 180 days and one year from discharge, stratified by different subgroups of the study population with interaction term P-values.

| **Subgroup** | **180 days**  **HR (95% CI)** | **P-value** | **Interaction**  **P-value** | **One year**  **HR (95% CI)** | **P-value** | **Interaction**  **P-value** |
| --- | --- | --- | --- | --- | --- | --- |
| **Sex** | | | | | | |
| Male | 0.67 (0.52-0.85) | **0.001** | 0.761 | 0.73 (0.59-0.91) | **0.005** | 0.507 |
| Female | 0.71 (0.52-0.97) | **0.031** | Reference | 0.84 (0.63-1.11) | 0.218 | Reference |
| **Age** | | | | | | |
| 18-30 years | 0.78 (0.47-1.28) | 0.323 | Reference | 0.89 (0.57-1.40) | 0.612 | Reference |
| 31-40 years | 0.57 (0.34-0.95) | **0.030** | 0.405 | 0.71 (0.46-1.09) | 0.117 | 0.523 |
| 41-50 years | 0.63 (0.42-0.96) | **0.029** | 0.511 | 0.65 (0.44-0.95) | **0.025** | 0.271 |
| 51-60 years | 0.65 (0.45-0.92) | **0.015** | 0.568 | 0.72 (0.52-0.99) | **0.046** | 0.463 |
| >60 years | 0.82 (0.53-1.26) | 0.361 | 0.873 | 0.94 (0.64-1.39) | 0.770 | 0.816 |
| **Inpatient characteristics** | | | | | | |
| Voluntarily admitted | 0.65 (0.50-0.85) | **0.001** | Reference | 0.75 (0.59-0.94) | **0.013** | Reference |
| Involuntarily admitted | 0.74 (0.55-0.99) | **0.045** | 0.496 | 0.83 (0.64-1.09) | 0.179 | 0.538 |
| Length of stay  ≤ 14 days | 0.94 (0.61-1.47) | 0.798 | Reference | 0.96 (0.65-1.44) | 0.857 | Reference |
| Length of stay  15-30 days | 0.70 (0.44-1.12) | 0.134 | 0.368 | 0.79 (0.52-1.19) | 0.255 | 0.515 |
| Length of stay  >30 days | 0.73 (0.54-1.00) | **0.047** | 0.353 | 0.87 (0.65-1.15) | 0.328 | 0.707 |
| **Subtype** | | | | | | |
| No paranoid schizophrenia | 0.67 (0.52-0.88) | **0.004** | Reference | 0.84 (0.66-1.06) | 0.148 | Reference |
| Paranoid schizophrenia | 0.69 (0.52-0.91) | **0.009** | 0.813 | 0.70 (0.54-0.90) | **0.006** | 0.326 |
| **Psychiatric comorbidity** | | | | | | |
| No anxiety disorder | 0.65 (0.52-0.82) | **<0.001** | Reference | 0.73 (0.59-0.89) | **0.002** | Reference |
| Anxiety disorder | 0.76 (0.53-1.09) | 0.139 | 0.475 | 0.89 (0.64-1.24) | 0.487 | 0.278 |
| No cannabis use disorder | 0.70 (0.58-0.86) | **<0.001** | Reference | 0.79 (0.66-0.95) | **0.012** | Reference |
| Cannabis use disorder | 0.42 (0.20-0.89) | **0.023** | 0.306 | 0.39 (0.20-0.75) | **0.004** | 0.204 |
| No substance use disorder | 0.67 (0.54-0.82) | **<0.001** | Reference | 0.75 (0.62-0.91) | **0.004** | Reference |
| Substance use disorder | 0.76 (0.48-1.20) | 0.239 | 0.589 | 0.81 (0.54-1.22) | 0.315 | 0.617 |
| No major depressive disorder | 0.67 (0.53-0.83) | **<0.001** | Reference | 0.72 (0.59-0.89) | **0.001** | Reference |
| Major depressive disorder | 0.73 (0.50-1.07) | 0.105 | 0.668 | 0.93 (0.67-1.31) | 0.687 | 0.182 |
| **Prior psychiatric hospitalizations** | | | | | | |
| 0-5 prior hospitalizations | 0.68 (0.41-1.11) | 0.122 | 0.402 | 0.78 (0.52-1.20) | 0.258 | 0.534 |
| 6-15 prior hospitalizations | 0.55 (0.39-0.76) | **<0.001** | 0.053 | 0.64 (0.47-0.87) | **0.004** | 0.088 |
| 16-25 prior hospitalizations | 0.62 (0.39-0.99) | **0.043** | 0.220 | 0.72 (0.48-1.07) | 0.104 | 0.328 |
| >25 prior hospitalizations | 0.86 (0.62-1.19) | 0.366 | Reference | 0.92 (0.68-1.25) | 0.589 | Reference |
| **Years since first psychiatric admission** | | | | | | |
| ≤10 years | 0.68 (0.56-0.82) | **<0.001** | Reference | 0.77 (0.65-0.91) | **0.003** | Reference |
| 11-25 years | 0.66 (0.47-0.93) | **0.018** | 0.750 | 0.71 (0.53-0.97) | **0.030** | 0.542 |
| >25 years | 0.67 (0.50-0.89) | **0.006** | 0.774 | 0.77 (0.59-1.01) | 0.054 | 0.731 |
| **Total number antipsychotics ever collected** | | | | | | |
| 0-2 antipsychotics | 0.74 (0.54-1.03) | 0.072 | Reference | 0.81 (0.60-1.08) | 0.144 | Reference |
| 3-5 antipsychotics | 0.62 (0.46-0.83) | **0.001** | 0.452 | 0.73 (0.56-0.95) | **0.018** | 0.667 |
| >5 antipsychotics | 0.74 (0.48-1.12) | 0.154 | 0.980 | 0.80 (0.55-1.16) | 0.236 | 0.957 |
| **Antipsychotics collected within 100-180 days before admission^a^** | | | | | | |
| No long-acting injectable antipsychotics | 0.71 (0.56-0.89) | **0.003** | Reference | 0.83 (0.67-1.02) | 0.076 | Reference |
| Long-acting injectable antipsychotics | 0.64 (0.45-0.89) | **0.009** | 0.650 | 0.66 (0.48-0.90) | **0.008** | 0.211 |
| No clozapine | 0.77 (0.61-0.97) | **0.029** | Reference | 0.84 (0.68-1.04) | 0.100 | Reference |
| Clozapine | 0.52 (0.38-0.72) | **<0.001** | 0.058 | 0.62 (0.46-0.84) | **0.002** | 0.094 |
| **Other pharmacological agents collected within 100 days before admission** | | | | | | |
| No antidepressants before | 0.72 (0.56-0.92) | **0.010** | Reference | 0.80 (0.64-1.01) | 0.059 | Reference |
| Antidepressants before | 0.63 (0.47-0.85) | **0.002** | 0.543 | 0.72 (0.55-0.93) | **0.013** | 0.552 |
| No anxiolytics before | 0.70 (0.57-0.86) | **<0.001** | Reference | 0.78 (0.65-0.94) | 0.010 | Reference |
| Anxiolytics before | 0.57 (0.35-0.95) | **0.030** | 0.478 | 0.66 (0.42-1.05) | 0.081 | 0.526 |
| No benzodiazepines before | 0.67 (0.52-0.87) | **0.002** | Reference | 0.75 (0.60-0.95) | **0.015** | Reference |
| Benzodiazepines before | 0.69 (0.52-0.92) | **0.010** | 0.926 | 0.78 (0.60-1.02) | 0.067 | 0.931 |
| No mood stabilizers before | 0.69 (0.56-0.85) | **<0.001** | Reference | 0.77 (0.64-0.93) | **0.006** | Reference |
| Mood stabilizers before | 0.64 (0.40-1.01) | 0.057 | 0.863 | 0.73 (0.47-1.14) | 0.168 | 0.911 |
| **Family history in first-degree relatives** | | | | | | |
| No family history of psychotic disorders | 0.70 (0.57-0.86) | **<0.001** | Reference | 0.80 (0.66-0.96) | **0.015** | Reference |
| Family history of psychotic disorders | 0.56 (0.33-0.97) | **0.037** | 0.432 | 0.58 (0.35-0.96) | **0.034** | 0.193 |

Abbreviations: CI, confidence interval; ECT, electroconvulsive therapy; HR, hazard ratio.

^a^Antipsychotics in tablet form collected within 100 days before admission or long-acting injectable antipsychotics collected within 180 days before admission.
